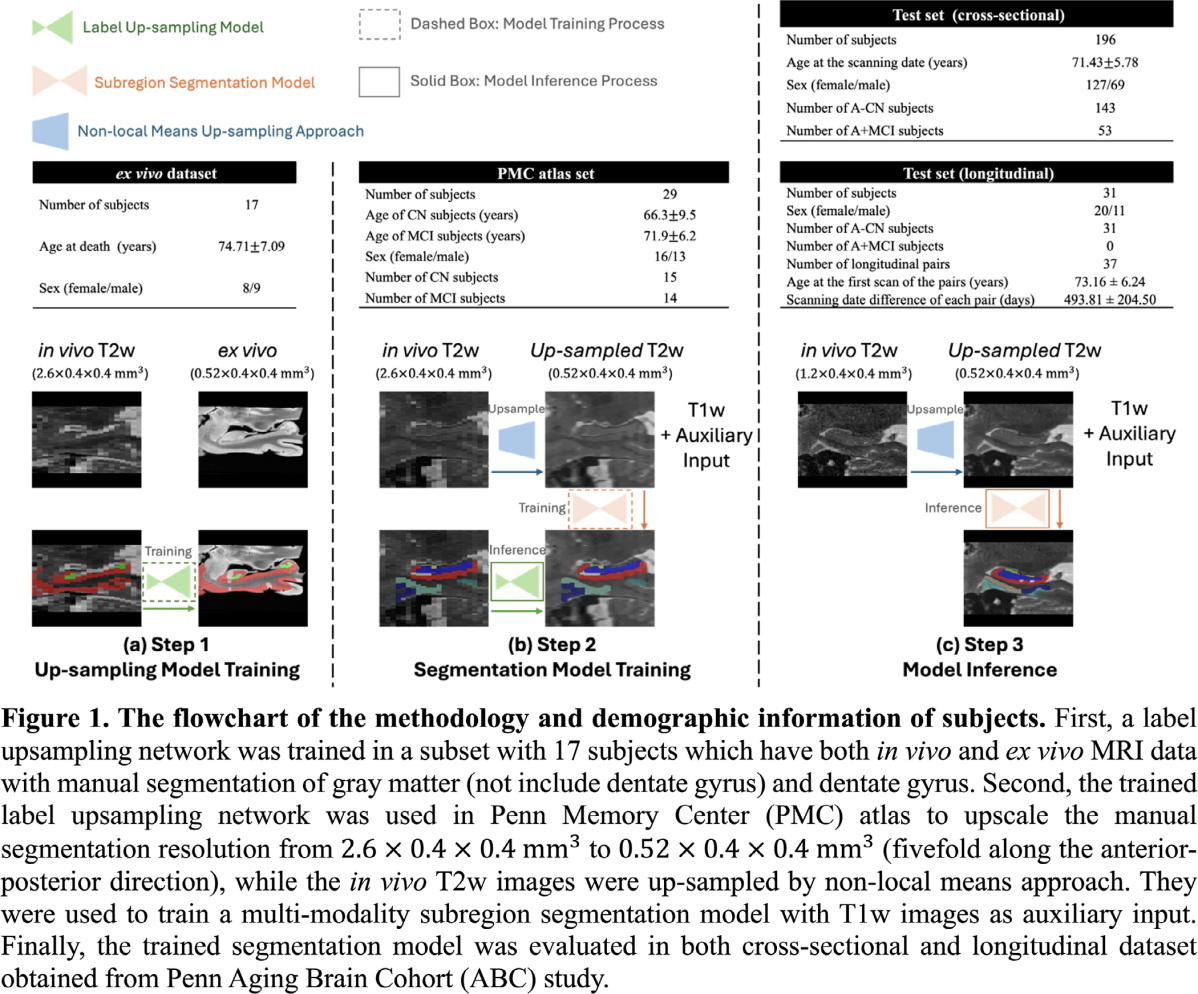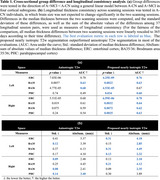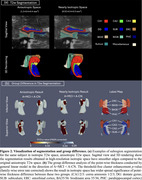# Segmentation of medial temporal lobe subregions in a nearly isotropic space using T2‐weighted MRI with anisotropic resolution

**DOI:** 10.1002/alz70856_105240

**Published:** 2026-01-07

**Authors:** Yue Li, Pulkit Khandelwal, Long Xie, Laura E.M. Wisse, Nidhi S. Mundada, Christopher A Brown, Emily McGrew, Amanda E Denning, Sandhitsu R. Das, David A. Wolk, Paul A. Yushkevich

**Affiliations:** ^1^ University of Pennsylvania, Philadelphia, PA, USA; ^2^ Siemens Healthineers, Princeton, NJ, USA; ^3^ Lund University, Lund, Sweden

## Abstract

**Background:**

Morphometry of medial temporal lobe (MTL) subregions in brain MRI is sensitive biomarker to Alzheimer's Disease and other related conditions. While T2‐weighted (T2w) MRI with high in‐plane resolution is widely used to segment hippocampal subfields due to its higher contrast in hippocampus, its lower out‐of‐plane resolution reduces the accuracy of subregion thickness measurements. To address this issue, we developed a nearly isotropic segmentation pipeline that incorporates image and label upsampling and high‐resolution segmentation in T2w MRI.

**Method:**

First, a high‐resolution atlas was created based on an existing anisotropic atlas derived from 29 individuals. Both T1‐weighted and T2w images in the atlas were upsampled from their original resolution to a nearly isotropic resolution using a non‐local means approach. Manual segmentations within the atlas were also upsampled to match this resolution using a UNet‐based neural network, which was trained on a cohort consisting of both high‐resolution *ex vivo* and low‐resolution anisotropic *in vivo* MRI with manual segmentations (Figure 1a). Second, a multi‐modality deep learning‐based segmentation model was trained within this nearly isotropic atlas (Figure 1b). This method was evaluated on independent sets, including cross‐sectional (*N* = 196) and longitudinal (*N* = 31) MRI scans, which were used for the group difference analysis (Amyloid+ mild cognitive impairment (A+MCI) vs. Amyloid‐ cognitively normal (A‐CN)) and longitudinal consistency analysis, respectively (Figure 1c).

**Result:**

Table 1(a) displays the group differences of cross‐sectional median thickness between A+MCI and A‐CN with age as covariate. The T2w segmentation in isotropic space achieved larger effect sizes in the predicted direction (A+MCI < A‐CN) and outperformed T2w anisotropic segmentation over most subregions. Table 1(b) shows the consistency analysis of longitudinal median thickness. When measured as the sum of absolute median thickness differences, the consistency of isotropic T2w segmentation outperformed that of anisotropic T2w segmentation over most subregions. Figure 2 shows the visualization of the segmentation and point‐wise group difference analysis at different resolutions, with isotropic T2w segmentation demonstrating a smoother surface and larger effect sizes than anisotropic T2w segmentation.

**Conclusion:**

Nearly isotropic subregion segmentation improved the accuracy of cortical thickness as an imaging biomarker for neurodegeneration in T2w MRI.